# Prevalence, antimicrobial resistance and genomic comparison of non-typhoidal salmonella isolated from pig farms with different levels of intensification in Yangon Region, Myanmar

**DOI:** 10.1371/journal.pone.0307868

**Published:** 2024-09-19

**Authors:** Nguyen Vinh Trung, Aung Zaw Moe, Hlaing May Than, Tran Thi Bich Chieu, A. S. Md Mukarram Hossain, Nguyen Trung Thanh, Huynh Xuan Yen, Phung Le Kim Yen, Nguyen Huu Nghia, Gemma G. R. Murray, Thiri Su Wai, Min Thein Maw, Hnin Thidar Myint, Ye Tun Win, James Wood, Guy Thwaites, Duncan J. Maskell, Alexander W. Tucker, Ngo Thi Hoa

**Affiliations:** 1 Oxford University Clinical Research Unit, Ho Chi Minh City, Vietnam; 2 Faculty of Veterinary Medicine, College of Agriculture, Can Tho University, Can Tho, Vietnam; 3 Livestock Breeding and Veterinary Department, Ministry of Agriculture, Livestock and Irrigation, Nay Pyi Taw, Myanmar; 4 Department of Veterinary Medicine, University of Cambridge, Cambridge, United Kingdom; 5 Cancer Research UK Manchester Institute Cancer Biomarker Centre, University of Manchester, Alderley Park, Macclesfield, United Kingdom; 6 Department of Genetics, Evolution and Environment, University College London, London, United Kingdom; 7 Nuffield Department of Medicine, Centre for Tropical Medicine, University of Oxford, Oxford, United Kingdom; 8 University of Melbourne, Melbourne, Australia; 9 Microbiology Department and Center for BioMedicine Research, Pham Ngoc Thach University of Medicine, Ho Chi Minh City, Vietnam; University of Illinois Urbana-Champaign College of Veterinary Medicine, UNITED STATES OF AMERICA

## Abstract

In Myanmar, where backyard, semi-intensive, and intensive pig (*Sus scrofa domesticus*) farming coexist, there is limited understanding of the zoonotic risks and antimicrobial resistance (AMR) associated with these farming practices. This study was conducted to investigate the prevalence, AMR and genomic features of *Salmonella* in pig farms in the Yangon region and the impact of farm intensification to provide evidence to support risk-based future management approaches. Twenty-three farms with different production scales were sampled for two periods with three sampling-visit each. Antimicrobial susceptibility tests and whole-genome sequencing were performed on the isolates. The prevalence of *Salmonella* was 44.5% in samples collected from backyard farms, followed by intensive (39.5%) and semi-intensive farms (19.5%). The prevalence of multi-drug resistant isolates from intensive farms (45/84, 53.6%) was higher than those from backyard (32/171, 18.7%) and semi-intensive farms (25/161, 15.5%). Among 28 different serovars identified, *S*. Weltevreden (40; 14.5%), *S*. Kentucky (38; 13.8%), *S*. Stanley (35, 12.7%), *S*. Typhimurium (22; 8.0%) and *S*. Brancaster (20; 7.3%) were the most prevalent serovars and accounted for 56.3% of the genome sequenced strains. The diversity of *Salmonella* serovars was highest in semi-intensive and backyard farms (21 and 19 different serovars, respectively). The high prevalence of globally emerging *S*. Kentucky ST198 was detected on backyard farms. The invasive-infection linked typhoid-toxin gene (*cdtB*) was found in the backyard farm isolated *S*. Typhimurium, relatively enriched in virulence and AMR genes, presented an important target for future surveillance. While intensification, in terms of semi-intensive versus backyard production, maybe a mitigator for zoonotic risk through a lower prevalence of *Salmonella*, intensive production appears to enhance AMR-associated risks. Therefore, it remains crucial to closely monitor the AMR and virulence potential of this pathogen at all scales of production. The results underscored the complex relationship between intensification of animal production and the prevalence, diversity and AMR of *Salmonella* from pig farms in Myanmar.

## Introduction

Non-typhoid salmonellosis is a common, potentially life-threatening, foodborne zoonotic disease in both developed and developing countries [1]. However, the true prevalence of non-typhoid salmonellosis is frequently underestimated due to asymptomatic, self-limiting infections and the lack of testing and surveillance program. The global burden of NTS gastroenteritis was estimated at almost 94 million cases each year, of which 86% are foodborne infections and 57% were in East Asia [1]. *Salmonella* is also associated with systemic invasive disease with an estimated 535,000 cases in 2017 and a mean case fatality rate of 14.5% [2]. Pork is the second most frequent source of human salmonellosis after poultry in Europe [3]. In Asian countries, pig (*Sus scrofus domsticus*)-derived *Salmonella* commonly reported serovars were Derby, Typhimurium, Rissen, Anatum, Weltevreden, and Enteritidis [4]. These are also the most common serovars isolated from humans in Asia [5]. A previous study demonstrated correlations between pig-derived *S*. Derby in pigs and human illness in this region [6].

There is limited data available on the prevalence of *Salmonella* in pig farms or slaughterhouses in Myanmar [7]. Similarly, data on human salmonellosis in Myanmar is sparse even in the case of blood-borne *Salmonella* [8, 9]. Pork is the second most commonly consumed meat in Myanmar and it has been estimated to account for approximately 25% of protein supply [10]. Similar to other developing countries worldwide, most pigs in Myanmar are farmed in backyard-scale farms with fewer than 10 pigs, which are characterized by a very low level of investment and lack of effective biosecurity [11]. A study with 44 medium and larger-scale pig farms in the Yangon Region showed that even though the pig industry was active, the number of pigs reared in these farms was low, with an average of 149 pigs /farm/ year. These findings suggest the presence of a small number of larger farms among a base of mid-sized farms [12]. This survey revealed evidence of the intensification of pig production in Myanmar, including uptake of commercial pig feeds and improved, imported, genetics in a move away from slower-growing local pig breeds. As a consequence, pig farming practices in Myanmar, and their intensification, could pose a risk for *Salmonella* infections in humans.

This study investigated the prevalence, phenotypic and genotypic antimicrobial resistance (AMR), and genomic features of *Salmonella* isolated from pig farms with different levels of intensification in Yangon, Myanmar, providing evidence to support risk-based future management approaches.

## Materials and methods

### Study location, farm recruitment and sample collection

A total of 23 farms from Yangon Region, Myanmar, were recruited. Pig farms representing three discrete pig production scales in Myanmar (backyard, semi-intensive and intensive) were recruited based on the willingness of the farmers to participate and the long-term expectation of keeping pigs through to slaughter age (5–7 months). A scoping study, led by Myanmar’s Livestock Breeding and Veterinary Department (LBVD), identified 3 townships characterized by a predominance of one or other of these farm scales. Farms were then identified through a preliminary field census of farming activity in village tracts, again led by LBVD. *Backyard production* farms were located in one suburban township (SD), these farms primarily consisted of farms with fewer than 10 pigs and were characterized by low levels of investment and limited bio-containment measures. *Semi-intensive production* farms were identified in two rural townships (TK and HL) and typically housed 10–30 pigs and were oriented towards commercial production. *Intensive production* farms were housed between 2,000 to 7,000 pigs and located in the government-designated ‘Livestock Intensive Zone’ of HL township (the terms SD, TK, and HL have been used for confidentiality of the studied townships). The farms were studied in two periods spanning between December 2016 and May 2020. The base-line/first-sampling and the follow-up/second-sampling periods were from December 2016 to September 2017 and July 2019 to May 2020, respectively. In each period, sampling was conducted across the three seasons: winter (October to January), summer (February to May), and rainy season (June to September).

Before the collection of any samples or data, study information and informed consent procedures were completed with farm owners and managers by a trained local field team from LBVD. In each of the farm visits, farm environmental samples were collected including boot-swabs (1 to 5 faecal boot-swabs) and drainage sample (one sample). For boot swab sampling, each sample was collected on each pen within a farm. New plastic over-boots and disposable gloves were used to collect each sample to prevent cross-contamination. The cotton-boot covers were used over the plastic-boot covers and were made damp with 50 ml of sterile de-ionized water to facilitate sample collection via walking on the pen floor as described previously [13]. Then the pair of cotton-boot covers were removed to store at 4°C before transfered to the laboratory. Farm drainage sampling involved collecting 15–20 ml of wastewater from three different points and stored in Falcon tubes. These containers were recapped, labeled, and then also stored at 4°C before transferring to the laboratory.

All samples were transported to the Veterinary Diagnostic Laboratory at LBVD in Yangon in a foam box with ice packs (4°C-10°C) within 5 hours post sample collection for microbiological culture and isolation.

### Sample processing, *Salmonella* isolation and antimicrobial susceptibility testing

A modification of the ISO 6579:2002 (Annex D) method was used for *Salmonella* isolation as described previously [14]. Briefly, the samples were processed with: (a) 225 ml of buffered peptone water (Oxoid, UK) for pre-enrichment at 37°C for 18 hours; (b) following by plating the pre-enriched culture (100 ul) onto modified semi-solid Rappaport–Vassiliadis medium (Oxoid; UK) at 41°C for 24 hours; and then (c) plating on Rambach agar (Chromagar, France) at 37°C for 24 hours for confirmation.

A maximum of three suspected *Salmonella* colonies were selected from each of the Rambach agar plates, equivalent to each of the samples positive for *Salmonella*. Each colony was confirmed by both slide agglutination with polyvalent *Salmonella* O (PSO) and polyvalent *Salmonella* H (PSH) antisera (ThermoFisher, USA).

Each selected *Salmonella* isolate was subjected to antimicrobial susceptibility testing by the disc diffusion method [15]. The antimicrobial susceptibility breakpoints were interpreted in accordance with the Clinical and Laboratory Standards Institute (CLSI) guidelines for Enterobacteriaceae [16]. Nine tested antimicrobials were chloramphenicol (30 μg), ceftazidime (30 μg), ceftriaxone (30 μg), amoxicillin/clavulanic acid (30 μg), ciprofloxacin (5 μg), trimethoprim/sulfamethoxazole (1.25/23.75 μg), nalidixic acid (30 μg), gentamicin (10 μg) and ampicillin (10 μg) (Oxoid, UK). Quality controls for susceptibility testing and bacterial identification were performed weekly according to the CLSI guidelines [16]. Strains with an antimicrobial intermediate susceptibility result were considered resistant. A multi-drug resistant (MDR) strain was defined as a strain that was resistant to at least three different classes of antimicrobials. The strains were shipped to the Oxford University Clinical Research Unit’s laboratory and then identified with Matrix-assisted laser desorption/ionization-time of flight (MALDI-TOF) Mass Spectrometry (Bruker, USA). A farm was defined as positive for *Salmonella* if at least one *Salmonella* strain was isolated from any sample. A sample was defined as positive for MDR *Salmonella* if at least one MDR strain was detected from the sample.

### Whole-genome sequencing analysis including *in silico* identification of antimicrobial resistance and virulence genes

Within each sample, up to a maximum of 3 *Salmonella* isolates, each with a unique antimicrobial-resistance phenotype pattern were selected for whole genome sequencing (WGS). DNA was extracted using the Wizard Genomic DNA purification kit (Promega, USA) in accordance with the manufacturer’s instructions. Sequencing was performed at the sequencing facility at the Biochemistry Department of the University of Cambridge using the Illumina NextSeq 500 (Illumina, USA) with paired-end reads of length 150 bp.

An assembly pipeline using SPAdes v3.12.1 was conducted to generate *de novo* genome assemblies [17]. These assemblies were annotated with Prokka [18]. The output was used for the pan-genome pipeline using Roary [19] to construct the core gene alignment. SNPs in the core gene alignment were identified by using snp-sites (https://github.com/sanger-pathogens/snp-sites) and an approximate maximum-likelihood tree was reconstructed using FastTree version 2.1.3 [20]. The pairwise SNP distance between isolates was calculated using the tool snps-dists (https://github.com/tseemann/snp-dists). Phylogenetic trees were visualized using iTOL [21]. Multi-Locus Sequence Typing (MLST) and serovar identification was achieved using SISTR on the *de novo* assemblies [22]. We employed ARIBA [23] to identify antimicrobial resistance and virulence genes using the ResFinder database [24] and virulence database [25], respectively.

### Data analysis

Differences in prevalence and AMR proportions were compared using the Chi-square test or Fisher’s Exact Test. A p-value ≤ 0.05 was considered statistically significant. A 95% confidence interval for the estimated proportion was calculated using the following formula: $\bar{x}$ *± 1*.96* σ/√n, where $\bar{x}$ is the sample mean, *σ* is the population standard deviation and *n* is the sample size. Bonferroni correction was also used to adjust the p-values in the comparison of resistance and virulence genes across different groups.

Discriminant analysis of principal components (DAPC) was used to compare the AMR gene and virulence gene profiles of different study groups [26]. ‘Survey’, ‘epicalc’, ‘ggplot2’ and ‘adegenet’ packages were used to perform the statistical analyses and visualization using R statistical software (http://www.r-project.org).

### Ethical approval

The project was approved by the ethics committee of the University of Cambridge (HBREC.2015.20). Written informed consent was obtained from all farmers prior to participation in the study. All the studies and activities with detailed proposals were approved by LBVD prior to implementation and field site access.

## Results

### Sample collection from different farms

Samples were collected from 23 farms for two sampling periods. Initially, there were 18 farms with 2 intensive, 10 semi-intensive, and 6 backyard farms and each was visited three times in three seasons for each sampling period. After the first visit, one farm dropped out and was replaced and then visited to collect samples from the second visit. A total of 54 farm visits in 19 recruited farms during this sampling period, resulting in 328 samples. In the second period, 13 of 19 original farms remained. Only four new farms were identified to replace the drop-out farms. However, not all of these 17 farms kept pigs for the entire duration of the second sampling period due to the emergence of African Swine Fever disease in Myanmar, resulting in 45 farm visits and 172 samples during this period (S1 Table).

### Description of farm demographic and management factors

Of the 23 farms included in this study, the median number of pigs per farm exhibited notable variations: 6 for backyard farms, 15 for semi-intensive farms, and 5827 for intensive farms (S2 Table). While borehole/well water was the primary drinking water source for pigs on all semi-intensive and intensive farms, 75.0% of backyard farms relied on river water. A similar pattern emerged in terms of feed sources, with all semi-intensive and intensive farms opting for commercial feed, while 75.0% of backyard farms utilized kitchen leftovers/wastes. Most farms in the study also raised additional animals such as cattle, chickens, and ducks. However, biosecurity measures were notably lower in some backyard and semi-intensive farms, such as lack of boot bath/foot dip at the entrance, and casual footwears were not changed before entering the pig pens (S2 Table), compared to intensive ones. We also found that antimicrobial usage in the last 6 months was significantly higher in intensive and semi-intensive farms (100.0% and 76.9%, respectively) compared to backyard farms (25.0%). Furthermore, vaccine (Classical Swine Fever/Foot and/or Mouth Disease/Porcine Reproductive and/or Respiratory Syndrome/Porcine Circovirus Type 2) usage was reported by 62.5% of backyard farms, 92.3% of semi-intensive farms, and 100.0% of intensive farms (S2 Table).

## Prevalence of *Salmonella* in farm visits and samples

All 23 farms were tested positive for *Salmonella* on at least one visit. Across the 99 farm visits where 500 samples were collected (S1 Table), *Salmonella* was detected in 65 farm visits (65.7%, 95% CI = 55.9% - 74.3%). *Salmonella*-positive samples were identified in 25/31 (83.3%), 29/56 (51.7%), and 11/12 (91.7%) of the farm visits at backyard, semi-intensive and intensive farms, respectively.

*Salmonella* was isolated from 147/500 samples (29.4%), combining all farm scales and sampling periods. The prevalence of *Salmonella*-positive samples was significantly higher in drainage samples (42/103, 40.8%) compared to bootswab samples (105/397, 26.4%) (*P* = 0.006) (Table 1).

**Table 1:** *Salmonella* prevalence in samples collected from pig farms in Yangon Region, Myanmar (2016–2020).

	Intensive		Semi-intensive		Backyard		Overall	
Variable	No. of positive samples /Total	Prevalence (95% CI)	No. of positive samples /Total	Prevalence (95% CI)	No. of positive samples /Total	Prevalence (95% CI)	No. of positive samples /Total	Prevalence (95% CI)
**All**	30/76	39.5 (28.4–50.6)	56/287	19.5 (14.9–24.1)	61/137	44.5 (36.2–52.8)	147/500	29.4 (25.4–33.3)
**Type of sample**								
Boot-swab	21/60	35.0 (23.5–46.5)	42/231	18.2 (13.2–23.2)	42/106	39.6 (30.3–48.9)	105/397	26.4 (22.2–30.6)
Drainage	9/16	56.3 (30.8–81.7)	14/56	25.0 (13.7–36.3)	19/31	61.3 (44.1–78.4)	42/103	40.8 (31.3–50.3)
**Study period**								
Baseline	11/40	27.5 (14.3–40.7)	23/180	12.8 (7.9–17.7)	40/108	37.0 (27.9–46.1)	74/328	22.6 (18.0–27.1)
Follow-up	19/36	52.8 (36.8–68.9)	33/107	30.8 (22.1–39.6)	21/29	72.4 (56.1–88.7)	73/172	42.4 (35.0–49.7)
**Season**								
Rainy	7/24	29.2 (11.6–46.8)	23/100	23.0 (14.8–31.2)	27/48	56.2 (42.2–70.3)	57/172	33.1 (26.2–40.0)
Summer	15/28	53.6 (34.0–73.2)	22/93	23.7 (15.0–32.3)	15/44	34.1 (20.1–48.1)	52/165	31.5 (24.7–38.3)
Winter	8/24	33.3 (14.6–52.1)	11/94	11.7 (5.2–18.2)	19/45	42.2 (27.8–56.7)	38/163	23.3 (16.6–30.1)

CI: Confidence interval

Between the sampling periods, irrespective of farming scale, the prevalence of *Salmonella*-positive samples was higher in the follow-up year (73/172, 42.4%) compared to that of the baseline year (74/328, 22.6%) (*P* = 5.8 x 10^−6^). This was also observed in both bootswab (48/127, 37.8% vs 57/270, 21.2%, p = 0.0006) and drainage samples (25/45, 55.6% vs 17/58, 29.3%, p = 0.01). Between farm scales, irrespective of sampling period and sample type, the prevalence of *Salmonella*-positive samples was highest in backyard farms (61/137, 44.5%), followed by intensive farms (30/76, 39.5%). Each of these was significantly higher than that of semi-intensive farms (56/287, 19.5%) (*P* = 1.3 x 10^−7^ and *P* = 4.8 x 10^−4^, respectively). We observed a lower *Salmonella* prevalence of samples collected in the winter season but that was not statistically significant (Table 1).

### Prevalence of antimicrobial resistant *Salmonella* isolates

A total of 416 *Salmonella* isolates were cultured from the 147 *Salmonella* positive samples, including 8 samples that yielded 1 isolate of *Salmonella*; 9 samples that yielded 2 isolates and 130 samples that yielded 3 isolates (S1 Table). Combining data to include all farm scales and both sampling periods, resistance to ampicillin (44.5%), nalidixic acid (38.5%), chloramphenicol (18.3%), trimethoprim/sulfamethoxazole (17.6%), ciprofloxacin (16.1%), gentamicin (10.8%), amoxicillin plus clavulanic acid (8.9%), ceftriaxone (8.4%), ceftazidime (7.0%) and MDR (24.5%) was detected amongst the 416 *Salmonella* isolates (Table 2). Resistance to 3^rd^ generation cephalosporins increased significantly by more than two-fold between sampling windows, for combined farm scales (ceftriaxone–baseline 10/212 (4.7%), follow-up 25/204 (12.3%); (*P* = 0.01), with intensive farms recording the highest prevalence across scales in the first sampling period. However, the prevalence increased in the follow-up sampling period for backyard and semi-intensive farms, such that the difference in prevalence between farm scales became less evident. While the prevalence of AMR for ciprofloxacin showed little variation between farm scales or sampling periods, the prevalence of antimicrobial resistant to gentamicin was significantly higher in the intensive farms but with no significant changes between sampling periods.

**Table 2:** Prevalence of AMR *Salmonella* among 416 isolates from pig farms in Yangon Region, Myanmar (2016–2020).

			Total number of isolates	Ampicillin	Ceftriaxone	Ceftazidime	Ciprofloxacin	Nalidixic acid	Chloramphenicol	Trimethoprim sulfamethoxazole	Amoxicillin clavulanate	Gentamicin	MDR*
**All isolates**			**416**	**185 (44.5)**	**35 (8.4)**	**29 (7.0)**	**67 (16.1)**	**160 (38.5)**	**76 (18.3)**	**73 (17.6)**	**37 (8.9)**	**45 (10.8)**	**102 (24.5)**
**Study period**													
		Baseline	212	96 (45.3)	10 (4.7)	9 (4.2)	37 (17.5)	98 (46.2)	30 (14.2)	33 (15.6)	22 (10.4)	18 (8.5)	47 (22.2)
		Follow-up	204	89 (43.6)	25 (12.3)	20 (9.8)	30 (14.7)	62 (30.4)	46 (22.5)	40 (19.6)	15 (7.4)	27 (13.2)	55 (27.0)
**Farm scale**													
		Intensive	84	71 (84.5)	12 (14.3)	6 (7.1)	12 (14.3)	36 (42.9)	34 (40.5)	34 (40.5)	15 (17.9)	31 (36.9)	45 (53.6)
		Semi-intensive	161	48 (29.8)	11 (6.8)	11 (6.8)	21 (13.0)	42 (26.1)	26 (16.1)	24 (14.9)	9 (5.6)	8 (5.0)	25 (15.5)
		Backyard	171	66 (38.6)	12 (7.0)	12 (7.0)	34 (19.9)	82 (48.0)	16 (9.4)	15 (8.8)	13 (7.6)	6 (3.5)	32 (18.7)
**Study period and farm scale**													
	**Baseline**	Intensive	32	32 (100)	5 (15.6)	3 (9.4)	6 (18.8)	19 (59.4)	18 (56.2)	15 (46.9)	13 (40.6)	10 (31.2)	25 (78.1)
		Semi-intensive	69	21 (30.4)	2 (2.9)	2 (2.9)	10 (14.5)	32 (46.4)	7 (10.1)	12 (17.4)	6 (8.7)	5 (7.2)	13 (18.8)
		Backyard	111	43 (38.7)	3 (2.7)	4 (3.6)	21 (18.9)	47 (42.3)	5 (4.5)	6 (5.5)	3 (2.7)	3 (2.7)	9 (8.1)
	**Follow-up**	Intensive	52	39 (75.0)	7 (13.5)	3 (5.8)	6 (11.5)	17 (32.7)	16 (30.8)	19 (36.5)	2 (3.8)	21 (40.4)	20 (38.5)
		Semi-intensive	92	27 (29.3)	9 (9.8)	9 (9.8)	11 (12.0)	10 (10.9)	19 (20.7)	12 (13.0)	3 (3.3)	3 (3.3)	12 (13.0)
		Backyard	60	23 (38.3)	9 (15)	8 (13.3)	13 (21.7)	35 (58.3)	11 (18.3)	9 (15.0)	10 (16.7)	3 (5.0)	23 (38.3)

*MDR: strain was resistant to at least three different classes of antimicrobials

While there was no significant difference in the overall prevalence of MDR *Salmonella* isolated in the baseline and follow-up period (22.2% and 27.0%), the detected prevalence was lower in the follow-up than in the baseline year for intensive farms (25/32, 78.1% *vs*. 20/52, 38.5%) (*P* = 0.9 x 10^−3^) and higher in backyard farms (9/111, 8.1% *vs*. 23/60, 38.3%) (*P =* 3.6 x 10^−6^). However, on combining data across the two sampling periods to identify any effect of farming scale, the prevalence of MDR isolates from intensive farms (45/84, 53.6%) was higher than those from backyard (32/171, 18.7%) (*P* = 2.8 x 10^−8^) and semi-intensive farms (25/161, 15.5%)(*P =* 1.0 x 10^−9^) (Table 2).

Among the 500 collected samples across farm scales and sampling periods, 56 (11.2%) were positive for MDR *Salmonella*. The prevalence of MDR *Salmonella* samples was significantly greater in the follow-up period (30/172, 17.4%) compared to the baseline (26/328, 7.9%) (*P* = 0.002) (S3 Table). Most of this change was explained by a large increase in sample MDR prevalence for backyard farms (baseline prevalence 6.5% (7/108) versus follow-up prevalence 32.1% (9/28) (*P* = 0.8 x 10^−3^, Fisher’s Exact Test). A similar but not statistically significant increase was seen for semi-intensive farms (baseline 4.4% (8/180), follow-up 10.2% (11/108)). The MDR sample prevalence remained high and similar on intensive farms at both sampling windows (baseline 27.5% (11/40), follow-up 27.7% (10/36).

### Serovar diversity of non-typhoidal *Salmonella*

**Fig 1:**
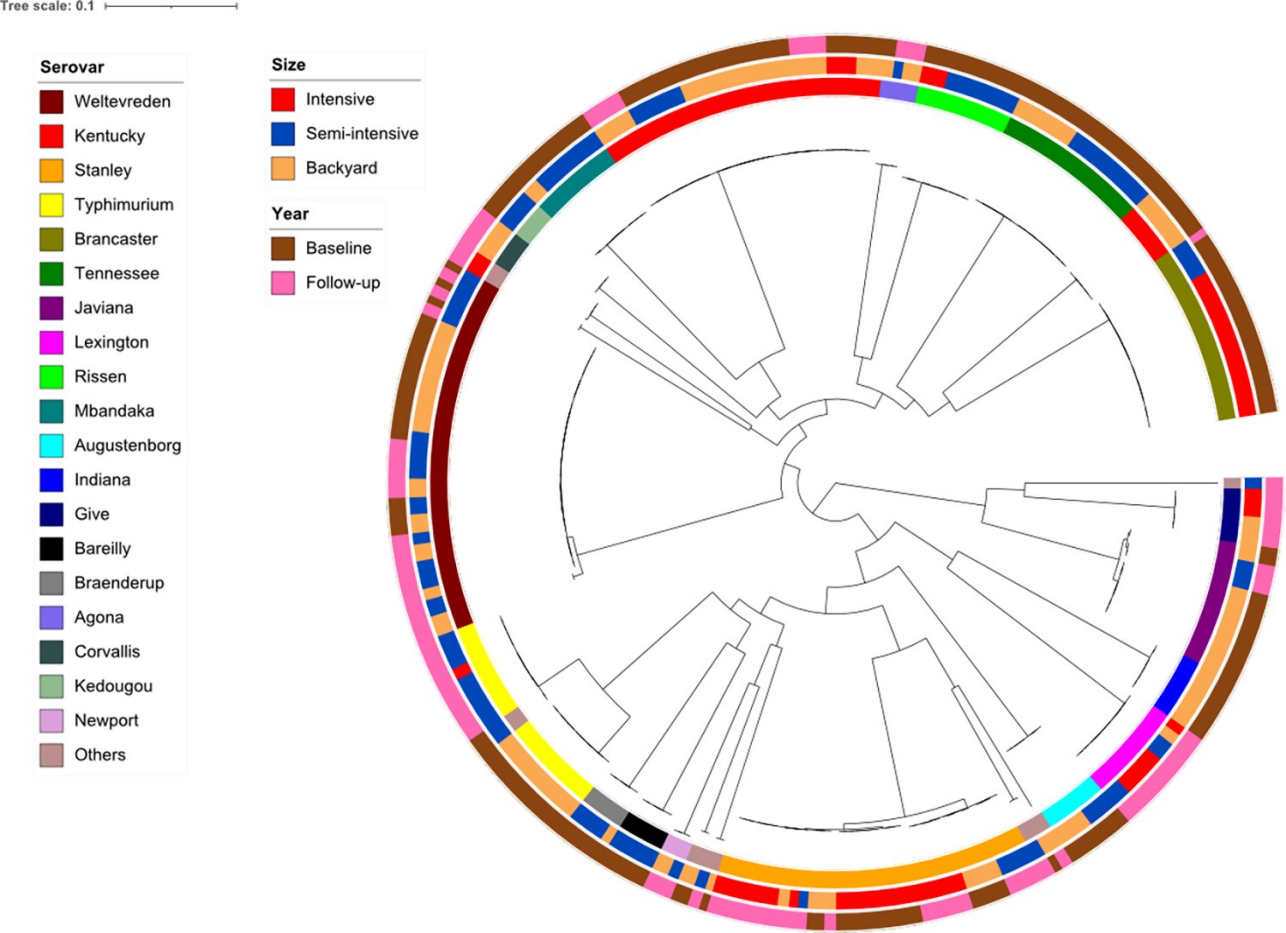
Circular maximum-likelihood core-gene phylogenetic tree of *Salmonella* isolated from pig farms in Yangon, Myanmar. The phylogenetic tree was reconstructed based on 162,062 SNPs in the core genome of 275 Salmonella isolates. Inner ring designates the serovars of the isolates (top 20 most common serovars). Middle ring designates the farm scale. Outer ring designates the study year.

Among the 416 strains that underwent phenotypic AMR characterisation, 275 strains were whole-genome sequenced. These strains originated from intensive (56), semi-intensive (100), and backyard (119) farms, spanning both the baseline (179) and follow-up (96) periods (S1 Table). The phylogeny reconstructed based on SNPs on the core genome suggested a diverse collection of *Salmonella* strains, exhibiting variations across both farm scales and sampling periods (Fig 1).

In total, 28 NTS serovars were *in-silico* identified and their numbers varied across the intensive (8 serovars, 9 STs), semi-intensive (21 serovars and STs) and backyard farms (19 serovars, 20 STs). The most prevalent serovars were *S*. Weltevreden (40; 14.5%), *S*. Kentucky (38; 13.8%), *S*. Stanley (35, 12.7%), *S*. Typhimurium (22; 8.0%) and *S*. Brancaster (20; 7.3%), which collectively accounted for 56.3% of the genome-sequenced strains (Fig 1 and S4 Table). Only four serovars, including three of the above, were detected across all farm scales (*S*. Kentucky, *S*. Stanley, *S*. Lexington and *S*. Typhimurium) (S4 Table). The most prevalent serovars, and their respective proportions, varied between farming scales: Stanley (22/56, 39.3%, 2 farms) for intensive farms; Weltevreden (19/100, 19%) for semi-intensive farms, 13 farms); and Kentucky (29/119, 24.4%, 8 farms) for backyard farms (S4 Table).

We identified 20 and 16 serovars in the baseline and follow-up periods, respectively. Only 8/28 (28.6%) serovars were detected in both periods (Fig 1 and S4 Table). Among the 13 farms sampled in both years, the same serovars detected in both periods were found on 4 farms. These included 2 backyard farms (*S*. Kentucky), one semi-intensive farm (*S*. Weltevreden) and one intensive farm (*S*. Stanley). However, among the 20 farms with more than one visit, the proportion of farms with the same serovar(s) found in at least 2 consecutive visits (in the same period) in intensive, semi-intensive and backyard farms was 50.0% (1/2), 41.7% (5/12) and 66.7% (4/6), respectively (S1 Fig).

Most *Salmonella* serovars are represented by a single sequence type (ST) except for *S*. Kentucky and *S*. Typhimurium (Fig 1). *S*. Kentucky, found mainly among backyard farms, included ST198, as the predominant ST shared by 32 isolates, and ST314, represented by only 6 isolates, found from a single backyard farm. Of the 22 *S*. Typhimurium isolates, found among all 3 farm types, eleven each were ST34 and ST36. The latter was also found only in a single backyard farm.

Although up to 3 isolates of each sample were chosen for analysis, we found that multiple isolates from the same sample belonged to the same serovar in the majority of cases (98/123 samples, 79.7%—S5 Table). Although there was no statistical difference, a greater range of serotypes present in a given sample was observed in backyard farms.

### Prevalence of antimicrobial resistance genes *in-silico* detected in *Salmonella* isolates

A total of 37 AMR genes were identified using WGS. Overall, chromosomal-encoded aminoglycoside acetyltransferase (*aac(6’)-Iaa*) was the most commonly detected gene (100%), followed by tetracycline (*tet*(A), 40.7%) and quinolone (*qnrS1*, 27.3%) resistance genes (Table 3). The gene *aac(6’)-Iaa* has been shown previously to be a cryptic gene [27–29]. Therefore, this gene was excluded from the calculation for the aminoglycoside AMR gene class.

**Table 3:** Frequency of antimicrobial resistance genes detected using whole genome sequencing data of 275 *Salmonella* isolates from pig farms in Yangon Region, Myanmar (2016–2020).

No.	Class	Gene	Number of positive isolates (%)	Number of isolates (genes grouped in antibiotic classes (%)
1	Tetracycline	*tet*(A)	112 (40.7)	127 (46.2)
2		*tet*(M)	15 (5.5)	
3		*tet*(B)	11 (4)	
4		*tet*(C)	4 (1.5)	
5	Aminoglycoside	*aac*(6’)-*Iaa*	275 (100)	124 (41.5)*
6		*aph*(3’’)-*Ib*	47 (17.1)	
7		*aph*(6’’)-*Id*	47 (17.1)	
8		*aph*(3’’)-*Ia*	34 (12.4)	
9		*aad*A7	32 (11.6)	
10		*aad*A1	31 (11.3)	
11		*aac*(3)-*IId*	22 (8)	
12		*aad*A2	17 (6.2)	
13		*aad*A2b	10 (3.6)	
14		*aad*A17	9 (3.3)	
15		*aac*(3)-*IId*	3 (1.1)	
16	Beta-lactam	*bla*TEM-1B	70 (25.5)	108 (39.3)
17		*bla*TEM-176	20 (7.3)	
18		*bla*TEM-1C	12 (4.4)	
19		*bla*CTX-M-14	3 (1.1)	
20		*bla*CTX-M-55	3 (1.1)	
21	Sulfonamide	*sul*1	45 (16.4)	85 (30.9)
22		*sul*2	29 (10.5)	
23		*sul*3	22 (8)	
24	Quinolone	*qnr*S1	75 (27.3)	81 (29.5)
25		*qnr*S2	5 (1.8)	
26		*qnr*D1	1 (0.4)	
27	Trimethoprim	*dfr*A14	24 (8.7)	47 (17.1)
28		*dfr*A12	23 (8.4)	
29	Phenicol	*flo*R	30 (10.9)	37 (13.5)
30		*cat*A2	14 (5.1)	
31		*cml*A1	3 (1.1)	
32	Macrolide	*mph*(A)	20 (7.3)	23 (8.4)
33		*mef*(B)	3 (1.1)	
34	Colistin	*mcr*-3	22 (8)	22 (8.0) **
35		*mcr*-1	1 (0.4)	
36	Fosfomycin	*fos*A7	13 (4.7)	13 (4.7)
37	Lincosamide	*lnu*(F)	9 (3.3)	9 (3.3)

* Excluded gene *aac’*(6’)-*Iaa*

** One strain co-carrying both *mcr*-1 and *mcr*-3

**Fig 2:**
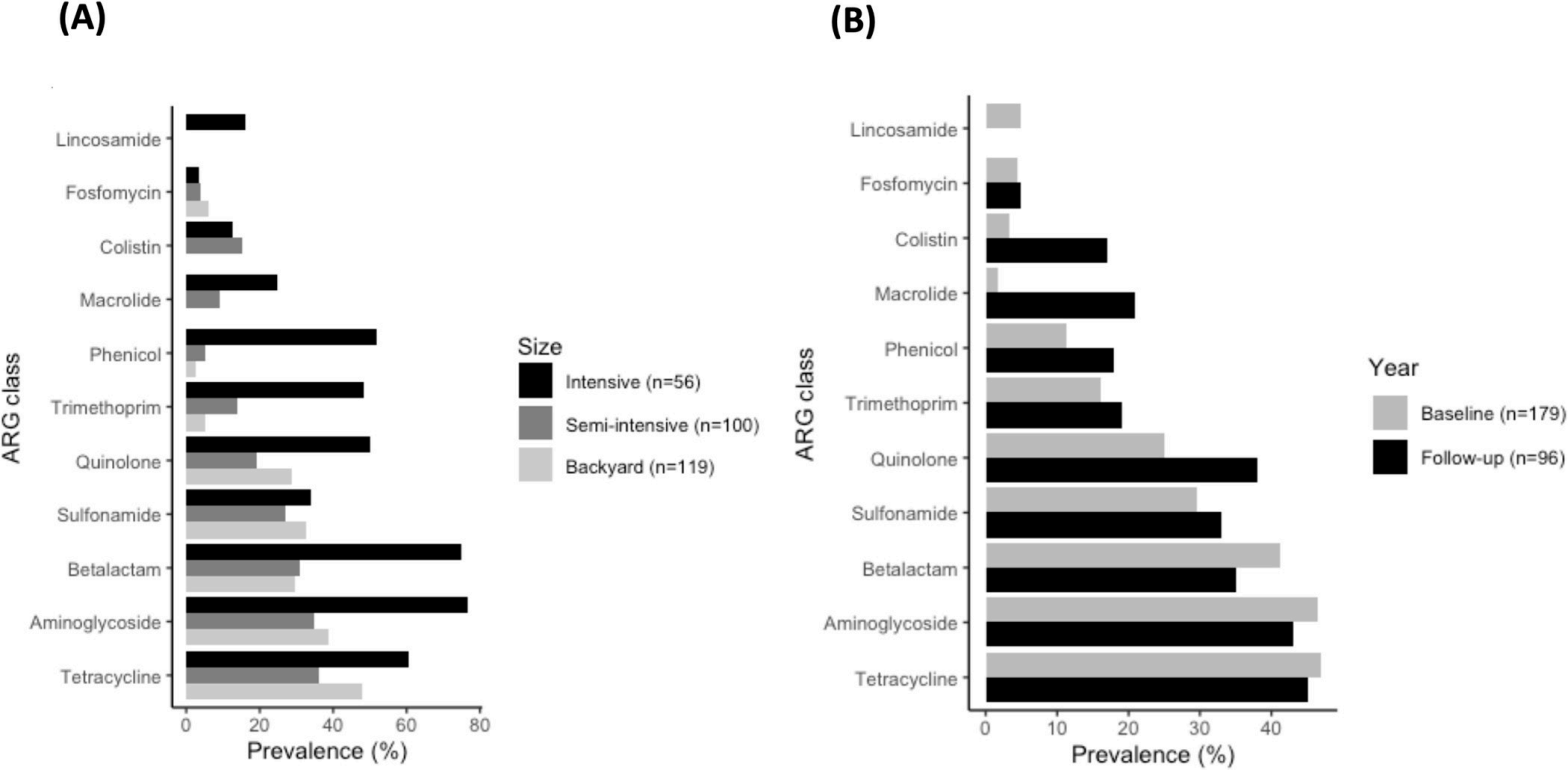
Prevalence of AMR genes detected in *Salmonella* isolates from pig farms in Yangon Region, Myanmar (2016–2020), grouped by antibiotic classes in (A) different farm scales and (B) study periods.

Collectively, isolates from intensive and semi-intensive farms contained AMR genes conferring resistance to a wider range of antimicrobials than those from backyard farms (11 and 10 vs. 8 classes of antibiotics, respectively). Higher proportions of isolates from intensive farms carried AMR genes encoding for resistance to the tested antibiotics (grouped by classes), with the exception of fosfomycin and colistin (Fig 2A). AMR genes for colistin (*mcr*-1, *mcr*-3), lincosamide (*lnu*(F)) and macrolide (*mef*(B), *mph*(A)) were only detected in intensive and/or semi-intensive farms (Fig 2A). *Salmonella* from intensive farms carried a median of 7 AMR genes, while strains from other farm scales carried a median of 1 and 2 AMR genes. (S2A Fig). The DAPC analysis of AMR gene profiles indicated that strains from intensive farms were the most distinct, while those from semi-intensive and backyard farm scales were more similar to each other (S2B Fig). The prevalence of AMR genes to quinolones, macrolides and colistin in *Salmonella* was higher in the follow-up period (Fig 2B). There was also a greater median number of AMR genes per isolate from all of the 3 farm scales in the follow-up periods (S3 Fig). Extended-spectrum beta-lactamase (ESBL) genes were found in only 6 *Salmonella* strains (2.2%), including 3 *S*. Kentucky (*bla*_CTX-M-14_) and 3 *S*. Give isolates (*bla*_CTX-M-55_), which were isolated from two backyard farms in the follow-up year.

The majority of the resistance phenotypes were explained by the presence of known AMR genes with a high accuracy (> 80%) (S6 Table).

### Prevalence of virulence genes detected in *Salmonella* isolates

Using WGS, a total of 114 virulence genes were detected, with 48 (42.1%) genes found in all isolates. These included fimbrial adherence determinants (*csg*ACEFG, *fim*DFH), non-fimbrial adherence determinants (sinH), secretion system *(inv*ABCEGHI, *prg*HJK, *spaOPQRS*, *ssa*DGHJKNOPRSV, *sse*ABG, *sip*AC, *org*ABC, *sic*AP, *ssc*AB) and Mg uptake (*mgt*C) (S7 Table). The presence of major virulence genes in *Salmonella* strains isolated from different farm scales shown limited variation, with the exception of a gene encoding typhoid toxins, the *cdtB* gene. The *cdtB* gene was detected in 17 (6.2%) isolates of 3 *S*. Javiana, *S*. Indiana and *S*. Give serovars. Among these, twelve were from three backyard farms, while the other five were from one intensive (two *S*. Give isolates) and one semi-intensive farm (three *S*. Javiana isolates). The median number of virulence genes carried by each strain was similar, ranging from 100 to 101 genes, across different farm scales (S2C Fig). The DAPC analysis of virulence gene profiles from all farm scales was indistinguishable (S2D Fig).

## Discussion

Although *Salmonella* is recognized globally as a foodborne zoonotic pathogen with many cases attributed to pigs and pig products, little is known about *Salmonella* prevalence in pig production systems in Myanmar. This study provides valuable information on the *Salmonella* prevalence and their phenotypic and genotypic AMR across different pig production scales in Myanmar’s Yangon Region–one of the country’s key pig production areas.

*Salmonella* was found on every farm in this study, with higher sample prevalence in the follow-up period compared to the baseline (42.4% versus 22.6%, Table 1). Sample prevalence increased in the second sampling period, approximately doubling for all farm scales, and reaching a notable 72.4% for backyard farms (Table 1). The factors underlying this general increase in sample prevalence over time are not immediately obvious. However, we noted that the use of commercial feed increased across all farm scales (S2 Table), which was reported to be linked with an increase in *Salmonella* infections [30]. In addition, the outbreaks of a viral infection (ASF) during the second sampling period might lead to an increase of bacterial carriage in pig herds as reported previously, in which *S*. *suis* infections was increased during the outbreaks of porcine reproductive and respiratory syndrome virus [31]. The overall sample prevalence (29.4%) was similar to those reported for China (24.1%) and Vietnam (27.7%) [32, 33]. We observed different sample prevalence across different farm intensification scales, with the highest prevalence in backyard (44.5%), followed by intensive (39.5%) and semi-intensive farms (19.5%). Typical backyard farming practices (S2 Table) such as using swill (kitchen and catering waste) feeding and the provision of surface water for drinking by pigs (ponds and rivers) could explain this high prevalence [34, 35]. A higher prevalence in intensive compared to semi-intensive farms could be due to their larger size with associated practical challenges in managing faecal contamination, as previously shown [36]. We observed that the prevalence of *Salmonella* was significantly higher in drainage samples (40.8%) compared to bootswab samples (26.4%). The high prevalence of *Salmonella* in drainage samples was also reported in a previous study in Vietnam [37]. Therefore, drainage samples could be effectively used for *Salmonella* surveillance in pig farms, especially in resource-limited settings.

We observed a different variation in *Salmonella* serovar between different farm scales. *Salmonella* Kentucky was the most common serovar (24.4%) in backyard farms. As a fitness advantage of *S*. Kentucky in the poultry gut was reported, its high prevalence was likely due to the common exposure to poultry in these farms (both live poultry and through kitchen waste as shown in S2 Table) [38]. Interestingly, the globally emerging *S*. Kentucky ST198 resistant to ciprofloxacin and extended-spectrum cephalosporins [39, 40] was also found in one backyard farm in this study. *Salmonella* Weltevreden was the most and second most prevalent serovar in semi-intensive and backyard farms, respectively and was reported as one of the most common serovars in pigs in Southeast Asia [41, 42]. *Salmonella* Stanley was the most prevalent serovar in intensive farms. This could be explained by these farms sourcing their pigs originally from Thailand (data not shown), where *Salmonella* Stanley is frequently identified in pigs [43]. Overall, the proportion of farms with the same serovar(s) found in at least 2 consecutive visits (around 2–3 months apart) was 50.0% and in all farm scales (S1 Fig). This finding indicates that *Salmonella* is maintained on farms, a likely consequence of continuously populated holding pens with incomplete cleaning and disinfection between batches of resident pigs. It also indicates that all production systems, irrespective of the scale of intensification, are exposed to new sources of incoming *Salmonella*. Further studies may identify whether and how these sources vary according to the level of intensification and associated farm inputs and exposures, including commercial diets versus kitchen waste, other livestock present on the farm, and overseas versus in-country sources of pigs.

The significant increase in MDR *Salmonella* prevalence in backyard farms between the 2016–2017 and 2019–2020 sampling periods (from 6.5% to 32.1%), a smaller increase for semi-intensive farms, and its high prevalence in both periods of intensive farms (around 27.0%) are notable (S3 Table). It is also worth noting that 8.4% and 16.1% of these *Salmonella* strains were also resistant to antibiotics classified into “Watch” group by the World Health Organization, including 3^rd^ generation of cephalosporins and ciprofloxacin [44]. Future studies could confirm and understand the drivers of this increase, which may include the usage of antimicrobials or medicated feed driving AMR development on-farm, or the introduction of MDR strains through insufficiently heated kitchen waste as pig feed, contaminated commercial pig feed, the purchase of subclinical carriers as replacement pigs, the use of contaminated surface water for drinking by pigs, or changes in carriage or shedding among other sources of faecal contamination of the pigs’ environment (human or other livestock) (S2 Table). Although comparable regional data indicated that the MDR *Salmonella* sample prevalence in intensive pig farms in the Yangon Region (21/76, 27.6%) was lower than that in China (16/27, 59.3%) [33], other factors influence the level of risk this presents to pigs, pig meat consumers, farm workers and veterinarians–for example differences in access to primary health care.

The number of detected clinically relevant AMR genes was low among isolates from backyard and semi-intensive scales but higher for isolates from intensive production (Fig 2A). This corroborated the observed higher prevalence of phenotypic AMR found among isolates from intensive production across a wide range of antibiotics (Table 2). The prevalence of ESBL NTS found in our study was similar to reports from Thailand (2.1%) but lower than in China (8/27, 29.6%) [7, 33]. ESBL NTS detected only in backyard farms justifies wider confirmation and further investigation of production practices and inputs that may be associated with this finding, especially because 3^rd^ and 4^th^ generation cephalosporins usage was not reported in backyard farms (unpublished data), and indicating alternative potential sources, particularly with the low level of ‘bio-containment’ in these backyard farms (S2 Table). In addition, the prevalence of NTS carried plasmid-mediated colistin resistance genes (*mcr*-1 and *mcr*-3 in 22/275 strains (8.0%)) was higher and lower, respectively, than data from Thailand (1.0%) and China (18.5%) [7, 33]. In contrast to the presence of ESBL genes, *mcr-*1 and *mcr-*3 genes were only detected in intensive and semi-intensive farms. The presence of *mcr* genes suggests that colistin was used in the associated farms and gut bacteria could act as a reservoir for this gene. The fact that genes encoding resistance to colistin, lincosamides and macrolides were only detected in intensive and/or semi-intensive farms may reflect different antibiotic usage practices in different production systems. Strains of the most commonly isolated serovar, *S*. Weltevreden, and of commonly isolated serovars, *S*. Kentucky and *S*. Stanley, carried a low and slightly higher number of AMR genes (median = 1 versus 3, S4 Fig), respectively. Isolates of serovars *S*. I 1,4,[5],12:i:- and *S*. I 4,[5],12:i:-, and of other serovars, *S*. Agona, *S*. Typhimurium, *S*. Rissen, carried a higher number of AMR genes (median = 6 and 8–9), respectively (S4 Fig).

Limited variation in the frequency of major virulence genes across the farm scales was detected and the ‘classic’ virulence factors (e.g. *spv*, *stn*, *bfp*, *pef*, *sef*, *fli*) [45] were not found. Isolates of *S*. Typhimurium, *S*. I 1,4,[5],12:i:- and *S*. I 4,[5],12:i:- carried the highest number of virulence genes (median = 107–109) (S4 Fig). In our study these were relatively more prevalent on semi-intensive and backyard farms, collectively constituting 12% and 9.2% of sequenced isolates, but only 1.8% of sequenced isolates from intensive farms. The typhoid toxins gene, *cdtB* gene, was detected in 17 (6.2%) isolates from different farm scales with the majority of strains from backyard farms. Previous studies have shown that the *cdtB* gene was linked to isolates implicated in the human bloodstream and invasive infections [46, 47]. This suggests an exposure risk to *cdtB*-carrying *Salmonella* among farmers in Myanmar with the possibility, in turn, of human-derived sources presenting a risk for infection of pigs, through inadequate sanitation or contaminated water sources–more typically associated with smaller scale pig production.

*S*. Typhimurium strains carrying a high number of both AMR and virulence genes, found in all farm scales and among the top five most common serovars in semi-intensive and backyard farms, is an important serovar to target for surveillance and control in pig farms in Myanmar. Human-originated Paratyphi B isolates were also detected in one semi-intensive and two backyard farms, indicating potential cross-contamination with human waste, and further highlighting the complexity of pig-human *Salmonella* epidemiology for the different production systems. *Salmonella* Paratyphi and *Salmonella* Typhi have been reported as significant contributors to human febrile bloodstream infections in Myanmar [9].

We acknowledge the limitations of this study. We were able to include only a small number of farms in the region, with just two intensive farms. Recruitment of smaller farms was limited by logistical and cost constraints associated with sample collection and transport. Recruitment of intensive farms was severely restricted by biosecurity concerns from managers. This resulted in a limited number of samples and restricted our power to undertake more detailed comparisons of *Salmonella* characteristics between different farm scales and over time. Our study was restricted to the Yangon Region of Myanmar for the logistical and cost constraints noted above, so our findings may not be representative of the wider farming systems in the country and caution must be taken in extrapolating our findings. Furthermore, the high drop-out rate, associated with poor market conditions and exacerbated by African Swine Fever outbreaks globally and regionally, may affect the direct comparisons between the two periods but should still reflect the outcomes of those farming scales. Nevertheless, by recruiting farms from all three production scales and using a whole-genome sequencing approach, this study provides comprehensive data on the prevalence, AMR, serotype diversity and virulence of *Salmonella* isolated from different pig farm scales in Yangon Region–a leading pig production area of Myanmar. It provides much-needed evidence on which to base future studies of *Salmonella* prevalence and epidemiology in pig and human populations and provides insights into the potential significance of different pig production intensities.

## Conclusion

The results demonstrate the impact of intensification on the prevalence, AMR and genomic features of *Salmonella* from pig farms in Myanmar. While the *Salmonella* prevalence was high in both backyard and intensive farms, the MDR *Salmonella* prevalence was significantly higher in intensive farms. Genetic characteristics of *Salmonella* reveal the potential zoonotic risks of *Salmonella* infections, especially for *Salmonella* Typhimurium which was carrying a high number of both AMR and virulence genes.

## Supporting information

S1 Fig Detection of the same serovar in at least 2 consecutive visits across the studied farms (A) Number of sequenced isolates in each farm by different farm scales (B) Farm-by-farm breakdown of the proportion (%) of isolates available for sequencing from each visit. Blocks outlined in black indicate farm visits for which the same serovar(s) were found in at least 2 consecutive visits.
(TIF)

S2 Fig (A) The number of AMR genes found in each of 275 isolates of *Salmonella* spp (the solid line in each box plot represents the median value). (B) DAPC of AMR gene profiles of 275 *Salmonella* spp. (C) The number of virulence genes found in each of 275 isolates of *Salmonella* spp. (D) DAPC of virulence gene profiles of 275 *Salmonella* isolates. All data were categorized by farm scale.
(TIF)

S3 Fig Median number of AMR genes detected per isolate of the NTS collection for intensive, semi-intensive and backyard farm scales (A: baseline survey, B: follow-up survey).
(TIF)

S4 Fig Distribution of different NTS serovars isolated from pig farms and the corresponding number (median) of identified AMR genes and virulence genes. (TIF)

S1 Table Summary of NTS positive samples and sequenced isolates in each pig farm in Yangon Region, Myanmar (2016–2020) stratified by different sampling visits and study periods. Gray cells indicate NTS-negative samples. Black cells indicate unavailable isolates for sequencing.
(DOCX)

S2 Table Characteristics of 23 pig farms in Yangon Region, Myanmar (2016–2020). (DOCX)

S3 Table The prevalence of MDR *Salmonella* positive samples collected from pigs in different farm scales in Yangon Region, Myanmar (2016–2020). (DOCX)

S4 Table Distribution of different serovars among 275 isolates of *Salmonella* obtained from pig farms in Yangon Region, Myanmar (2016–2020). (DOCX)

S5 Table Distribution of intra-serovar homogeneity of *Salmonella* positive samples across different pig farm scales in Yangon Region, Myanmar (2016–2020). (DOCX)

S6 Table Association between the phenotype and the genotype of 275 sequenced *Salmonella* strains from pig farms in Yangon Region, Myanmar (2016–2020). (DOCX)

S7 Table Detected virulence genes in 275 *Salmonella* isolated from pig farms in Yangon Region, Myanmar (2016–2020). (DOCX)

S8 Table List of 275 *Salmonella* whole-genome sequences with associated data. (XLSX)
