# Supplementary figures and images for: Prevalence, antimicrobial resistance and genomic comparison of non-typhoidal salmonella isolated from pig farms with different levels of intensification in Yangon Region, Myanmar
Source: PLoS One. 2024 Sep 19;19(9):e0307868. doi: 10.1371/journal.pone.0307868 (PMC11412544; doi:10.1371/journal.pone.0307868)

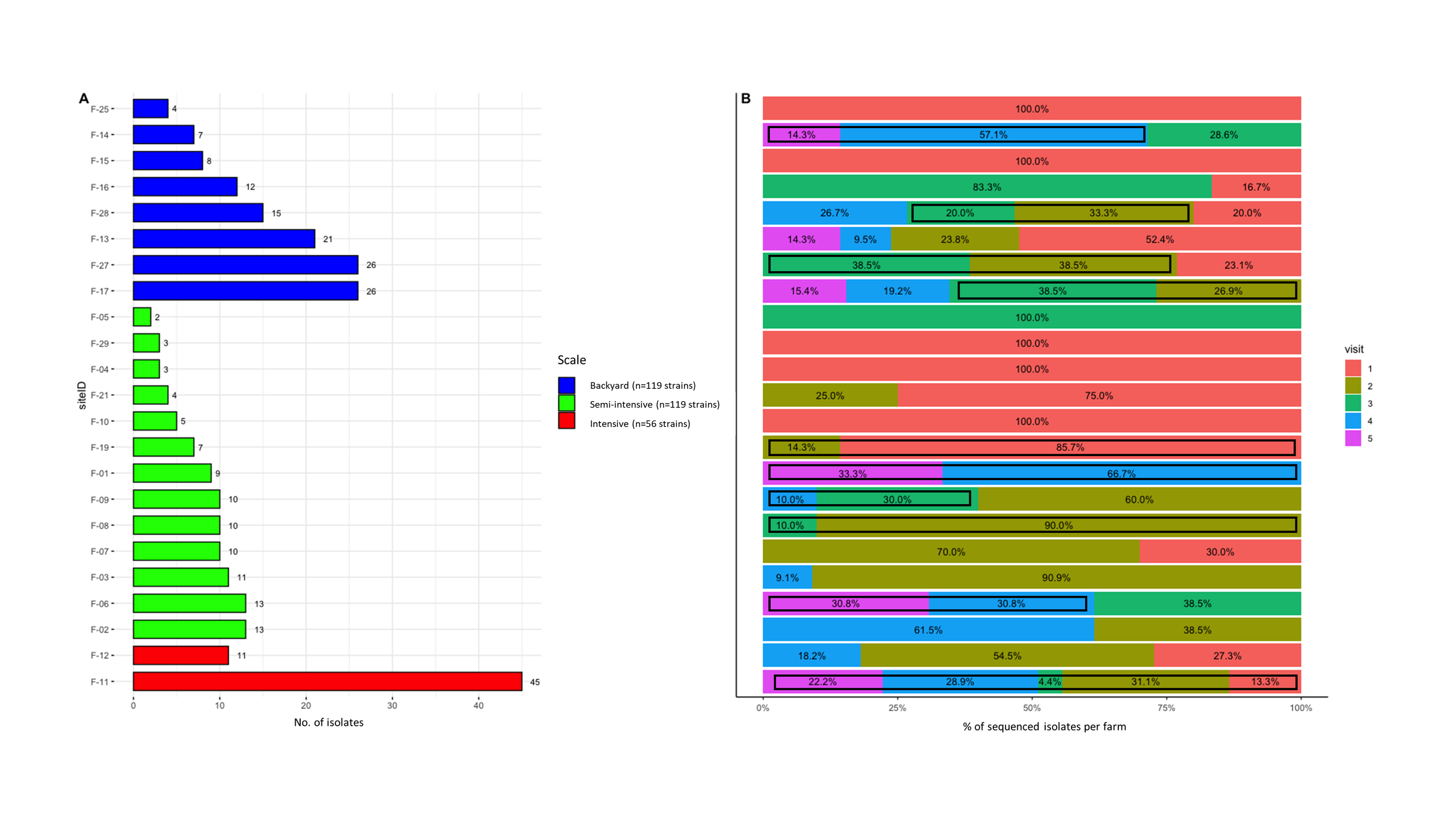

Supplement: S1 Fig — Blocks outlined in black indicate farm visits for which the same serovar(s) were found in at least 2 consecutive visits. (TIF) [file pone.0307868.s001.tif]

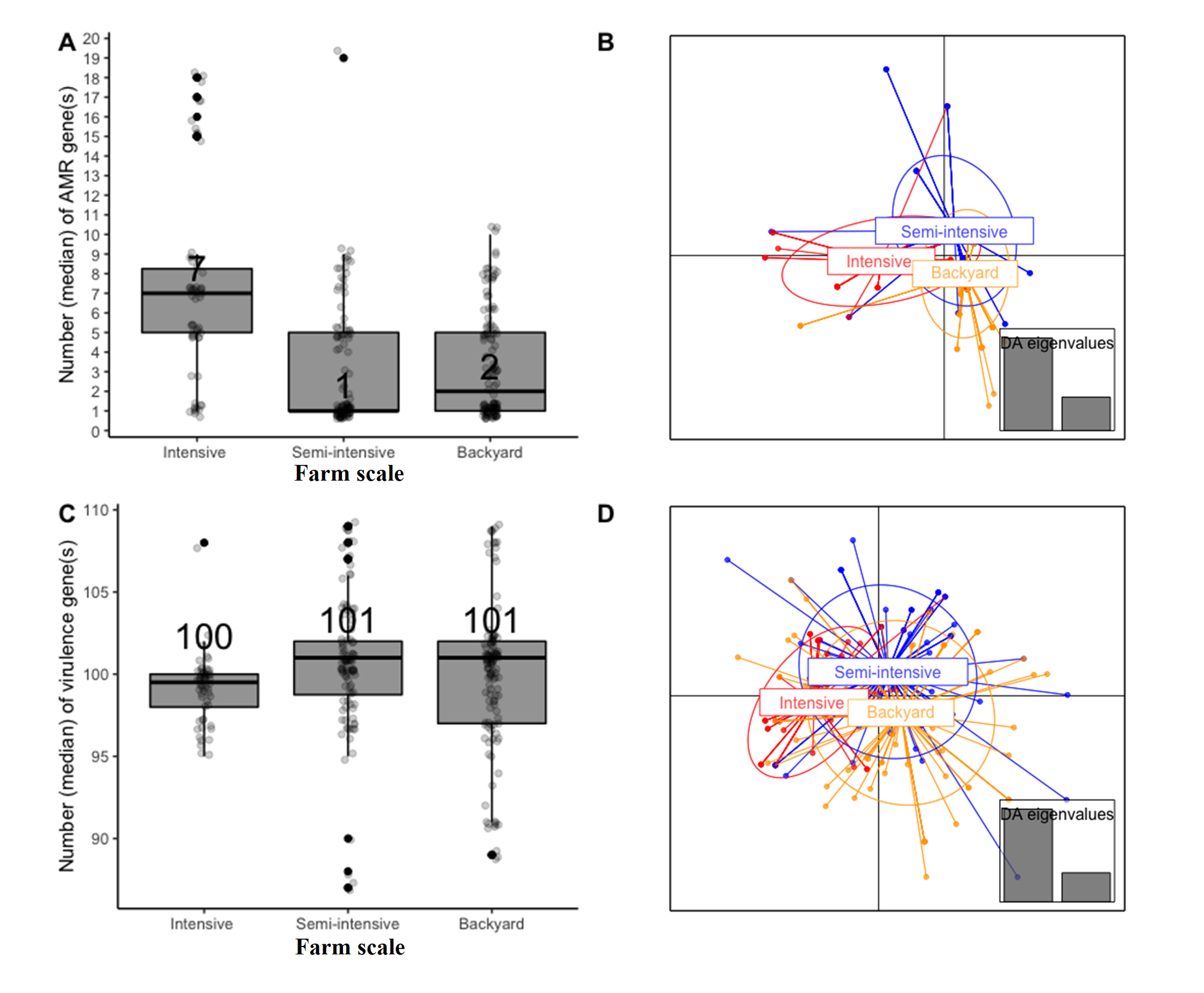

Supplement: S2 Fig — (A) The number of AMR genes found in each of 275 isolates of Salmonella spp (the solid line in each box plot represents the median value). (B) DAPC of AMR gene profiles of 275 Salmonella spp. (C) The number of virulence genes found in each of 275 isolates of Salmonella spp. (D) DAPC of virulence gene profiles of 275 Salmonella isolates. All data were categorized by farm scale. (TIF) [file pone.0307868.s002.tif]

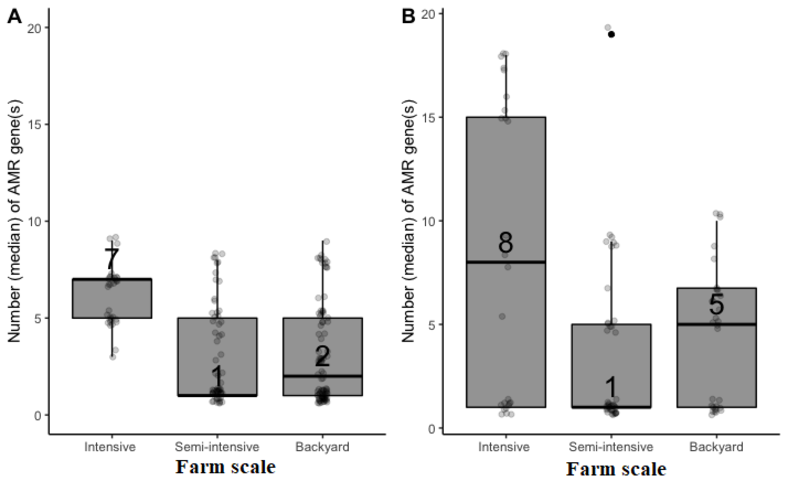

Supplement: S3 Fig — Median number of AMR genes detected per isolate of the NTS collection for intensive, semi-intensive and backyard farm scales (A: baseline survey, B: follow-up survey). (TIF) [file pone.0307868.s003.tif]

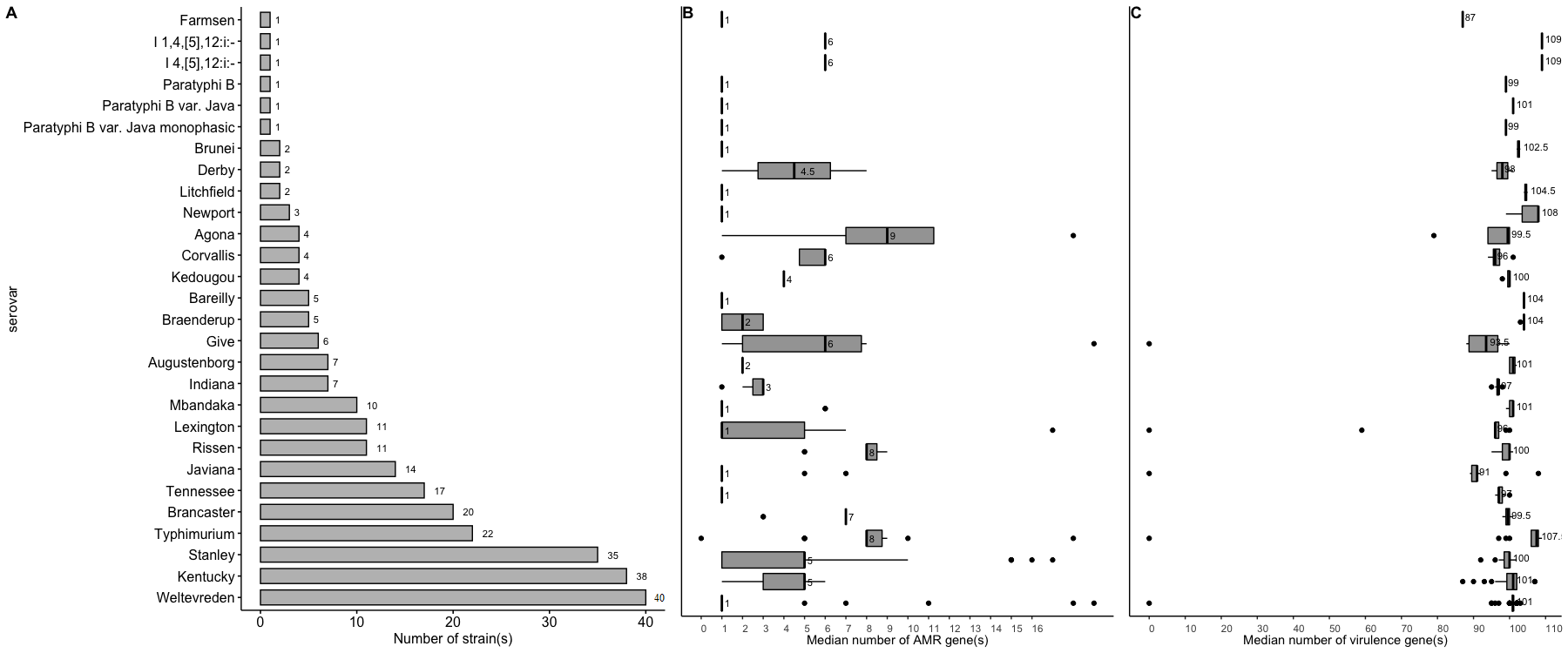

Supplement: S4 Fig — (TIF) [file pone.0307868.s004.tif]
