# Supplementary material for: Prevalence, antimicrobial resistance and genomic comparison of non-typhoidal salmonella isolated from pig farms with different levels of intensification in Yangon Region, Myanmar
Source: PLoS One. 2024 Sep 19;19(9):e0307868. doi: 10.1371/journal.pone.0307868 (PMC11412544; doi:10.1371/journal.pone.0307868)
Supplement: S1 Table — Gray cells indicate NTS-negative samples. Black cells indicate unavailable isolates for sequencing. (DOCX) [file pone.0307868.s005.docx]

|  |  | **No. of NTS positive samples (n=147)/ No. of collected samples (n=500)** | | | | | | |  | **No. of sequenced isolates (n=275)/No. of isolates (n=416)** | | | | | | |
| --- | --- | --- | --- | --- | --- | --- | --- | --- | --- | --- | --- | --- | --- | --- | --- | --- |
| **Farm scale** | **Farm ID** | **Baseline (74/328)** | | |  | **Follow-up (73/172)** | | |  | **Baseline (179/212)** | | |  | **Follow-up (96/204)** | | |
|  |  | **Visit 1** | **Visit 2** | **Visit 3** |  | **Visit 4** | **Visit 5** | **Visit 6** |  | **Visit 1** | **Visit 2** | **Visit 3** |  | **Visit 4** | **Visit 5** | **Visit 6** |
| ***Backyard*** | **F-13** | 4/6 | 2/6 | 0/6 |  | 1/2 | 1/2 | 2/2 |  | 11/12 | 5/6 |  |  | 2/3 | 3/3 | 0/6 |
|  | **F-14** | 0/6 | 0/6 | 1/6 |  | 2/2 | 1/3 | 2/2 |  |  |  | 2/2 |  | 4/5 | 1/3 | 0/6 |
|  | **F-15** | 4/6 |  |  |  |  |  |  |  | 8/10 |  |  |  |  |  |  |
|  | **F-16** | 2/6 | 0/6 | 5/6 |  |  |  |  |  | 2/4* |  | 10/14 |  |  |  |  |
|  | **F-17** | 0/6 | 3/6 | 4/6 |  | 3/4 | 2/2 | 1/2 |  |  | 7/9 | 10/12 |  | 5/7 | 4/6 | 0/3 |
|  | **F-25** |  |  |  |  | 2/2 |  |  |  |  |  |  |  | 4/6 |  |  |
|  | **F-27** | 3/6 | 4/6 | 5/6 |  |  |  |  |  | 6/8 | 10/10 | 10/15 |  |  |  |  |
|  | **F-28** |  | 1/6 | 2/6 |  | 2/2 | 2/2 | 0/2 |  |  | 3/3 | 5/6 |  | 3/6 | 4/6 |  |
| ***Semi-intensive*** | **F-01** | 0/6 | 0/6 | 0/6 |  | 3/6 | 2/6 | 0/5 |  |  |  |  |  | 6/9 | 3/4 |  |
|  | **F-02** | 0/6 | 2/6 | 0/6 |  | 4/6 | 0/4 | 1/3 |  |  | 5/6 |  |  | 8/11 |  | 0/2 |
|  | **F-03** | 0/6 | 4/6 | 0/6 |  | 1/6 | 0/6 | 1/6 |  |  | 10/12 |  |  | 1/3 |  | 0/3 |
|  | **F-04** | 1/6 | 0/6 | 0/6 |  |  |  |  |  | 3/3 |  |  |  |  |  |  |
|  | **F-05** | 0/6 | 0/6 | 1/6 |  |  |  |  |  |  |  | 2/3 |  |  |  |  |
|  | **F-06** | 0/6 | 0/6 | 2/6 |  | 3/6 | 2/4 | 1/4 |  |  |  | 5/6 |  | 4/9 | 4/5 | 0/1 |
|  | **F-19** |  |  |  |  | 3/4 | 2/4 | 0/6 |  |  |  |  |  | 6/9 | 1/6 |  |
|  | **F-29** |  |  |  |  | 1/2 |  |  |  |  |  |  |  | 3/3 |  |  |
|  | **F-07** | 1/6 | 3/6 | 0/6 |  |  |  |  |  | 3/3 | 7/9 |  |  |  |  |  |
|  | **F-08** | 0/6 | 3/6 | 1/6 |  | 0/2 |  |  |  |  | 9/9 | 1/3 |  |  |  |  |
|  | **F-09** | 0/6 | 2/6 | 1/6 |  | 1/4 | 0/6 | 1/4 |  |  | 6/6 | 3/3 |  | 1/3 |  | 0/3 |
|  | **F-10** | 2/6 | 0/6 | 0/6 |  | 0/2 | 0/2 | 2/3 |  | 5/6 |  |  |  |  |  | 0/6 |
|  | **F-21** |  |  |  |  | 2/2 | 1/2 | 2/2 |  |  |  |  |  | 3/6 | 1/3 | 0/6 |
| ***Intensive*** | **F-11** | 2/6 | 5/6 | 1/6 |  | 5/6 | 5/6 | 5/6 |  | 6/6 | 14/15 | 2/2 |  | 13/15 | 10/14 | 0/13 |
|  | **F-12** | 1/6 | 2/10 | 0/6 |  | 1/6 | 0/6 | 3/6 |  | 3/3 | 6/6 |  |  | 2/3 |  | 0/7 |

**One NTS positive sample (including one NTS isolate) was not available for sequencing*
