# Supplementary material for: Prevalence, antimicrobial resistance and genomic comparison of non-typhoidal salmonella isolated from pig farms with different levels of intensification in Yangon Region, Myanmar
Source: PLoS One. 2024 Sep 19;19(9):e0307868. doi: 10.1371/journal.pone.0307868 (PMC11412544; doi:10.1371/journal.pone.0307868)
Supplement: S2 Table — (DOCX) [file pone.0307868.s006.docx]

| **Variable** | **Backyard**  **(n=8)** | **Semi-intensive**  **(n=13)** | **Intensive**  **(n=2)** |
| --- | --- | --- | --- |
| No. pigs (median) (IQR) | 6 (3-9) | 15 (10-23) | 5827 (3428-8184) |
| Drinking water source for pigs*(No. farms) (%) |  |  |  |
| *Borehole/well* | 5 (62.5) | 13 (100) | 2 (100) |
| *River* | 6 (75.0) | 0 | 0 |
| Feed source for pigs*, (No. farms) (%) |  |  |  |
| *Commercial* | 6 (75.0) | 13 (100) | 2 (100) |
| *Kitchen leftover* | 6 (75.0) | 1 (7.7) | 0 |
| Presence of animals other than pig, (No. farms) (%) |  |  |  |
| *Cattle(s)* | 0 | 7 (53.8) | 0 |
| *Chicken(s)* | 5 (62.5) | 11 (84.6) | 2 (100) |
| *Duck(s)* | 1 (12.5) | 5 (38.5) | 0 |
| Change shoes/boots before entering pen, no. of farms (%) | 4 (50.0) | 8 (61.5) | 2 (100) |
| Foot bath/foot dip at entrance, no. of farms (%) | 3 (37.5) | 7 (53.8) | 2 (100) |
| Age at slaughter (days, median) (IQR) | 210 (173-270) | 150 (120-150) | 160 (150-165) |
| Any antimicrobials have been used**, (No. farms) (%) | 2 (25.0) | 10 (76.9) | 2 (100) |
| Any vaccines have been used**, (No. farms) (%) | 5 (62.5) | 12 (92.3) | 2 (100) |

IQR= Interquartile range

*Farm could have more than one answer

**In the last 6 months
