# Supplementary material for: Prevalence, antimicrobial resistance and genomic comparison of non-typhoidal salmonella isolated from pig farms with different levels of intensification in Yangon Region, Myanmar
Source: PLoS One. 2024 Sep 19;19(9):e0307868. doi: 10.1371/journal.pone.0307868 (PMC11412544; doi:10.1371/journal.pone.0307868)
Supplement: S3 Table — (DOCX) [file pone.0307868.s007.docx]

|  | **Baseline** |  | **Follow-up** |  | **Both years** |  |
| --- | --- | --- | --- | --- | --- | --- |
|  | **No. of samples** | **No. of MDR samples (%)** | **No. of samples** | **No. of MDR samples (%)** | **No. of samples** | **No. of MDR samples (%)** |
| All farm scales | 328 | 26 (7.9) | 172 | 30 (17.4) | 500 | 56 (11.2) |
| *Intensive* | 40 | 11 (27.5) | 36 | 10 (27.7) | 76 | 21 (27.6) |
| *Semi-intensive* | 180 | 8 (4.4) | 108 | 11 (10.2) | 288 | 19 (6.6) |
| *Backyard* | 108 | 7 (6.5) | 28 | 9 (32.1) | 136 | 16 (11.8) |
