# Supplementary material for: Prevalence, antimicrobial resistance and genomic comparison of non-typhoidal salmonella isolated from pig farms with different levels of intensification in Yangon Region, Myanmar
Source: PLoS One. 2024 Sep 19;19(9):e0307868. doi: 10.1371/journal.pone.0307868 (PMC11412544; doi:10.1371/journal.pone.0307868)
Supplement: S4 Table — (DOCX) [file pone.0307868.s008.docx]

| **No.** | **Serovar** | **ST** | **Total (n=275)** | **Year** | |  | **Farm scale** | | |
| --- | --- | --- | --- | --- | --- | --- | --- | --- | --- |
|  |  |  |  | **Baseline (n=179)** | **Follow-up (n=96)** |  | **Intensive (n=56)** | **Semi-intensive (n=100)** | **Backyard (n=119)** |
| 1 | Weltevreden | ST365 | 40 (14.5) | 20 (11.2) | 20 (20.8) |  | 0 | 19 (19.0) | 21 (17.6) |
| 2 | Kentucky | Both STs | 38 (13.8) | 30 (16.8) | 8 (8.3) |  | 3 (5.4) | 6 (6.0) | 29 (24.4) |
|  |  | ST198 | 32 (11.6) | 24 (13.4) | 8 (8.3) |  | 3 (5.4) | 6 (6.0) | 23 (19.3) |
|  |  | ST314 | 6 (2.2) | 6 (3.4) | 0 |  | 0 | 0 | 6 (5.0) |
| 3 | Stanley | ST29 | 35 (12.7) | 15 (8.4) | 20 (20.8) |  | 22 (39.3) | 5 (5.0) | 8 (6.7) |
| 4 | Typhimurium | Both STs | 22 (8.0) | 11 (6.1) | 11 (11.5) |  | 1 (1.8) | 10 (10.0) | 11 (9.2) |
|  |  | ST34 | 11 (4.0) | 0 | 11 (11.5) |  | 1 (1.8) | 10 (10.0) | 0 |
|  |  | ST36 | 11 (4.0) | 11 (6.1) | 0 |  | 0 | 0 | 11 (9.2) |
| 5 | Brancaster | ST2133 | 20 (7.3) | 19 (10.6) | 1 (1) |  | 16 (28.6) | 4 (4.0) | 0 |
| 6 | Tennessee | ST319 | 17 (6.2) | 17 (9.5) | 0 |  | 0 | 10 (10.0) | 7 (5.9) |
| 7 | Javiana | ST1547 | 14 (5.1) | 11 (6.1) | 3 (3.1) |  | 0 | 3 (3.0) | 11 (9.2) |
| 8 | Lexington | ST1542 | 11 (4) | 0 | 11 (11.5) |  | 6 (10.7) | 4 (4.0) | 1 (0.8) |
| 9 | Rissen | ST469 | 11 (4) | 11 (6.1) | 0 |  | 3 (5.4) | 8 (8.0) | 0 |
| 10 | Mbandaka | ST413 | 10 (3.6) | 10 (5.6) | 0 |  | 0 | 8 (8.0) | 2 (1.7) |
| 11 | Augustenborg | ST1030 | 7 (2.5) | 7 (3.9) | 0 |  | 0 | 3 (3.0) | 4 (3.4) |
| 12 | Indiana | ST2040 | 7 (2.5) | 7 (3.9) | 0 |  | 0 | 0 | 7 (5.9) |
| 13 | Give | ST516 | 6 (2.2) | 0 | 6 (6.3) |  | 3 (5.4) | 0 | 3 (2.5) |
| 14 | Bareilly | ST909 | 5 (1.8) | 5 (2.8) | 0 |  | 0 | 5 (5.0) | 0 |
| 15 | Braenderup | ST22 | 5 (1.8) | 5 (2.8) | 0 |  | 0 | 4 (4.0) | 1 (0.8) |
| 16 | Agona | ST13 | 4 (1.5) | 1 (0.6) | 3 (3.1) |  | 0 | 1 (1.0) | 3 (2.5) |
| 17 | Corvallis | ST1541 | 4 (1.5) | 0 | 4 (4.2) |  | 0 | 0 | 4 (3.4) |
| 18 | Kedougou | ST1543 | 4 (1.5) | 4 (2.2) | 0 |  | 0 | 4 (4.0) | 0 |
| 19 | Newport | ST31 | 3 (1.1) | 0 | 3 (3.1) |  | 0 | 1 (1.0) | 2 (1.7) |
| 20 | Brunei | ST1794 | 2 (0.7) | 1 (0.6) | 1 (1) |  | 0 | 1 (1.0) | 1 (0.8) |
| 21 | Derby | ST40 | 2 (0.7) | 0 | 2 (2.1) |  | 2 (3.6) | 0 | 0 |
| 22 | Litchfield | ST214 | 2 (0.7) | 2 (1.1) | 0 |  | 0 | 0 | 2 (1.7) |
| 23 | Farmsen | ST2812 | 1 (0.4) | 0 | 1 (1) |  | 0 | 1 (1.0) | 0 |
| 24 | I 1.4.[5].12:i:- | ST34 | 1 (0.4) | 1 (0.6) | 0 |  | 0 | 1 (1.0) | 0 |
| 25 | I 4.[5].12:i:- | ST34 | 1 (0.4) | 1 (0.6) | 0 |  | 0 | 1 (1.0) | 0 |
| 26 | Paratyphi B | ST43 | 1 (0.4) | 0 | 1 (1) |  | 0 | 0 | 1 (0.8) |
| 27 | Paratyphi B var. Java | ST423 | 1 (0.4) | 0 | 1 (1) |  | 0 | 1 (1.0) | 0 |
| 28 | Paratyphi B var. Java monophasic | ST423 | 1 (0.4) | 1 (0.6) | 0 |  | 0 | 0 | 1 (0.8) |
