# Supplementary material for: Prevalence, antimicrobial resistance and genomic comparison of non-typhoidal salmonella isolated from pig farms with different levels of intensification in Yangon Region, Myanmar
Source: PLoS One. 2024 Sep 19;19(9):e0307868. doi: 10.1371/journal.pone.0307868 (PMC11412544; doi:10.1371/journal.pone.0307868)
Supplement: S5 Table — (DOCX) [file pone.0307868.s009.docx]

| **Farm production scale (*No. of samples)*** | **1 isolate/sample** | | **2 isolates/sample** | | **3 isolates/sample** | |
| --- | --- | --- | --- | --- | --- | --- |
|  | **No. of sample** | **No. of samples with same serovar (%)** | **No. of sample** | **No. of samples where both isolates are same serovar (%)** | **No. of sample** | **No. of samples where all 3 isolates are same serovar (%)** |
| *Intensive (n=22)* | 1 | 1 (100) | 8 | 6 (75.0) | 13 | 11 (84.6) |
| *Semi-intensive (n=46)* | 9 | 9 (100) | 20 | 15 (75.0) | 17 | 14 (82.4) |
| *Backyard (n=55)* | 10 | 10 (100) | 26 | 17 (65.4) | 19 | 15 (78.9) |
