# Supplementary material for: Prevalence, antimicrobial resistance and genomic comparison of non-typhoidal salmonella isolated from pig farms with different levels of intensification in Yangon Region, Myanmar
Source: PLoS One. 2024 Sep 19;19(9):e0307868. doi: 10.1371/journal.pone.0307868 (PMC11412544; doi:10.1371/journal.pone.0307868)
Supplement: S6 Table — (DOCX) [file pone.0307868.s010.docx]

|  | | **P+/G+** | **P+/G-** | **P-/G+** | **P-/G-** | | **Accuracy** |
| --- | --- | --- | --- | --- | --- | --- | --- |
| **Aminoglycosides** | |  |  |  |  | |  |
| Gentamicin | | 18 (39.1) | 12 (5.2) | 28 (60.9) | 217 (94.8) | | 0.85 |
| **Chloramphenicol** | |  |  |  |  | |  |
| Chloramphenicol | | 32 (86.5) | 14 (5.9) | 5 (13.5) | 224 (94.1) | | 0.93 |
| **Folate pathway inhibitors** | |  |  |  |  | |  |
| Co-trimoxazole* | | 18 (81.8) | 31 (12.3) | 4 (18.2) | 221 (87.7) | | 0.87 |
| **Fluoroquinolones**** | |  |  |  |  | |  |
| Ciprofloxacin | | 30 (90.9) | 18 (7.4) | 3 (9.1) | 224 (92.6) | | 0.92 |
| **β-lactams** | |  |  |  |  | |  |
| Ampicillin | | 88 (86.3) | 26 (15.0) | 14 (13.7) | 147 (85.0) | | 0.85 |
| Ceftriaxone | | 1 (16.7) | 9 (3.3) | 5 (83.3) | 260 (96.7) | | 0.95 |
| Ceftazidime | | 6 (100.0) | 15 (5.6) | 0 (0) | 254 (94.4) | | 0.95 |
| Phenotype (P); Genotype (G); presence (+); absence (−).  *Combination of a *sul* and a *dfr* gene  **Triple point mutations (*gyr*A-83, *gyr*A-87 and *par*C-80) | | | | |  |  |  |
| Accuracy was calculated by (P+/G+) + (P−/G−)/n | | | | |  |  |  |
