# Supplementary material for: Prevalence, antimicrobial resistance and genomic comparison of non-typhoidal salmonella isolated from pig farms with different levels of intensification in Yangon Region, Myanmar
Source: PLoS One. 2024 Sep 19;19(9):e0307868. doi: 10.1371/journal.pone.0307868 (PMC11412544; doi:10.1371/journal.pone.0307868)
Supplement: S7 Table — (DOCX) [file pone.0307868.s011.docx]

| **No.** | **Virulence type** | **Gene** | **Annotation** | **No. of positive strains (%)** |
| --- | --- | --- | --- | --- |
| 1 | Fimbrial adherence determinants | csgA | Curlin major subunit CsgA | 275 (100) |
| 2 |  | csgC | Curli assembly protein CsgC | 275 (100) |
| 3 |  | csgE | Curli production assembly/transport protein CsgE | 275 (100) |
| 4 |  | csgF | Curli production assembly/transport protein CsgF | 275 (100) |
| 5 |  | csgG | Curli production assembly/transport protein CsgG | 275 (100) |
| 6 |  | fimD | Usher protein FimD | 275 (100) |
| 7 |  | fimF | Type I fimbriae adaptor protein FimF | 275 (100) |
| 8 |  | fimH | Type I fimbriae minor fimbrial subunit FimH, adhesin | 275 (100) |
| 9 |  | csgB | Minor curlin subunit precursor, curli nucleator protein CsgB | 274 (99.6) |
| 10 |  | fimI | Fimbrial protein internal segment | 274 (99.6) |
| 11 |  | csgD | Curli fibers/thin aggregative fimbriae csgD | 273 (99.3) |
| 12 |  | fimC | Chaperone protein FimC | 273 (99.3) |
| 13 |  | bcfB | Bovine colonization factor bcfB | 270 (98.2) |
| 14 |  | bcfC | Bovine colonization factor bcfC | 270 (98.2) |
| 15 |  | bcfF | Bovine colonization factor bcfF | 270 (98.2) |
| 16 |  | bcfD | Bovine colonization factor bcfD | 269 (97.8) |
| 17 |  | bcfE | Bovine colonization factor bcfE | 268 (97.5) |
| 18 |  | steB | Secreted effector protein SteB | 267 (97.1) |
| 19 |  | bcfG | Bovine colonization factor bcfG | 260 (94.5) |
| 20 |  | bcfA | Bovine colonization factor bcfA | 251 (91.3) |
| 21 |  | steA | Secreted effector protein SteA | 217 (78.9) |
| 22 |  | lpfA | Long polar fimbria protein LpfA | 190 (69.1) |
| 23 |  | lpfB | Long polar fimbrial chaperone protein LpfB | 190 (69.1) |
| 24 |  | lpfE | Long polar fimbrial minor subunit LpfE, adhesin | 190 (69.1) |
| 25 |  | steC | Secreted effector kinase SteC | 179 (65.1) |
| 26 |  | lpfC | Long polar fimbrial usher protein LpfC | 175 (63.6) |
| 27 |  | lpfD | Long polar fimbrial protein LpfD | 118 (42.9) |
| 28 | Nonfimbrial adherence determinants | sinH | Intimin-like protein sinH | 275 (100) |
| 29 |  | misL | extracellular matrix adhesin, autotransport protein MisL | 264 (96) |
| 30 |  | ratB | Putative intestinal colonization factor ratB | 116 (42.2) |
| 31 |  | shdA | Putative intestinal colonization factor, AIDA autotransporter-like protein shdA | 7 (2.5) |
| 32 | Secretion system | invA | Type III secretion system major export apparatus protein InvA | 275 (100) |
| 33 |  | invB | Type III secretion system protein InvB | 275 (100) |
| 34 |  | invC | Type III secretion system ATPase InvC | 275 (100) |
| 35 |  | invE | Type III secretion system gatekeeper invE | 275 (100) |
| 36 |  | invG | Type III secretion system secretin invG | 275 (100) |
| 37 |  | invH | Type III secretion system pilotin invG | 275 (100) |
| 38 |  | invI | Type III secretion system stalk protein InvI | 275 (100) |
| 39 |  | orgA | Oxygen-regulated invasion protein OrgA | 275 (100) |
| 40 |  | orgB | Type III secretion system stator OrgB | 275 (100) |
| 41 |  | orgC | Putative cytoplasmic protein orgC | 275 (100) |
| 42 |  | prgH | Type III secretion system outer MS ring protein PrgH | 275 (100) |
| 43 |  | prgJ | Type III secretion system inner rod protein PrgJ | 275 (100) |
| 44 |  | prgK | Type III secretion system inner MS ring protein PrgK | 275 (100) |
| 45 |  | sicA | Type III secretion-associated chaperone sicA | 275 (100) |
| 46 |  | sicP | Type III secretion-associated chaperone sicP | 275 (100) |
| 47 |  | sipA | Type III secretion system effector SipA, actin polymerizing activity | 275 (100) |
| 48 |  | sipC | Type III secretion system hydrophilic translocator, pore protein SipC | 275 (100) |
| 49 |  | spaO | Type III secretion system C ring protein SpaO | 275 (100) |
| 50 |  | spaP | Type III secretion system minor export apparatus protein SpaP | 275 (100) |
| 51 |  | spaQ | Type III secretion system minor export apparatus protein SpaQ | 275 (100) |
| 52 |  | spaR | Type III secretion system minor export apparatus protein SpaR | 275 (100) |
| 53 |  | spaS | Type III secretion system export apparatus switch protein SpaS | 275 (100) |
| 54 |  | ssaD | Type III secretion system outer MS ring protein SsaD | 275 (100) |
| 55 |  | ssaG | Type III secretion system needle filament protein SsaG | 275 (100) |
| 56 |  | ssaH | Type III secretion system protein SsaH | 275 (100) |
| 57 |  | ssaJ | Type III secretion system inner MS ring protein SsaJ | 275 (100) |
| 58 |  | ssaK | Type III secretion system stator SsaK | 275 (100) |
| 59 |  | ssaN | Type III secretion system ATPase SsaN | 275 (100) |
| 60 |  | ssaO | Type III secretion system stalk protein SsaO | 275 (100) |
| 61 |  | ssaP | Type III secretion system needle length regulator SsaP | 275 (100) |
| 62 |  | ssaR | Type III secretion system minor export apparatus protein SsaR | 275 (100) |
| 63 |  | ssaS | Type III secretion system minor export apparatus protein SsaS | 275 (100) |
| 64 |  | ssaV | Type III secretion system major export apparatus protein ssaV | 275 (100) |
| 65 |  | sscA | Type III secretion system stator SsaK | 275 (100) |
| 66 |  | sscB | Type III secretion system chaperone sscB | 275 (100) |
| 67 |  | sseA | Type III secretion system chaperone sseA | 275 (100) |
| 68 |  | sseB | Type III secretion system effector SseB | 275 (100) |
| 69 |  | sseG | Type III secretion system effector SseG | 275 (100) |
| 70 |  | invJ | Type III secretion system needle length regulator InvJ | 274 (99.6) |
| 71 |  | prgI | Type III secretion system needle filament protein PrgI | 274 (99.6) |
| 72 |  | sipB | Type III secretion system hydrophilic translocator, pore protein SipB | 274 (99.6) |
| 73 |  | sopB/sigD | Type III secretion system effector SopB, phosphoinositide phosphatase | 274 (99.6) |
| 74 |  | ssaC | Type III secretion system secretin SsaC | 274 (99.6) |
| 75 |  | ssaE | Pathogenicity island 2 type III secretion system specific chaperone SsaE | 274 (99.6) |
| 76 |  | ssaI | Type III secretion system inner rod protein SsaI | 274 (99.6) |
| 77 |  | ssaM | Type III secretion system protein SsaM | 274 (99.6) |
| 78 |  | ssaQ | Type III secretion system C ring protein SsaQ | 274 (99.6) |
| 79 |  | ssaT | Type III secretion system minor export apparatus protein SsaT | 274 (99.6) |
| 80 |  | sseD | Type III secretion system hydrophilic translocator, pore protein SseD | 274 (99.6) |
| 81 |  | sseE | Type III secretion system effector SseE | 274 (99.6) |
| 82 |  | pipB | Type III secretion system effector PipB | 273 (99.3) |
| 83 |  | sifA | Type III secretion system effector SifA, N-terminal SKIP-binding domain, C-terminal guanine nucleotide exchange factor activity | 273 (99.3) |
| 84 |  | ssaU | Type III secretion system export apparatus switch protein SsaU | 273 (99.3) |
| 85 |  | invF | Type III secretion system regulatory protein InvF | 272 (98.9) |
| 86 |  | sseJ | Type III secretion system effector SseJ, glycerophospholipid:cholesterol acyltransferase | 272 (98.9) |
| 87 |  | sipD | Type III secretion system hydrophilic translocator, needle tip protein SipD | 271 (98.5) |
| 88 |  | ssaL | Type III secretion system gatekeeper SsaL | 271 (98.5) |
| 89 |  | spiC/ssaB | Type III secretion system protein SsaB | 270 (98.2) |
| 90 |  | sopE2 | Type III secretion system effector SopE2, guanine nucleotide exchange factor | 266 (96.7) |
| 91 |  | sifB | Secreted effector protein SifB | 265 (96.4) |
| 92 |  | sopD | Type III secretion system effector SopD | 265 (96.4) |
| 93 |  | sptP | Type III secretion system effector SptP, tyrosine phosphatase and GTPase-activating protein | 265 (96.4) |
| 94 |  | sseC | Type III secretion system hydrophilic translocator, pore protein SseC | 265 (96.4) |
| 95 |  | sseF | Type III secretion system effector SseF | 264 (96) |
| 96 |  | sseL | Deubiquitinase SseL | 257 (93.5) |
| 97 |  | pipB2 | Type III secretion system effector PipB2 | 251 (91.3) |
| 98 |  | sopA | Type III secretion system effector SopA, E3 ubiquitin ligase | 222 (80.7) |
| 99 |  | avrA | Type III secretion system effector AvrA, acetyltransferease | 213 (77.5) |
| 100 |  | sseK1 | Type III secretion system translocated effectors sseK1 | 180 (65.5) |
| 101 |  | sopD2 | Secreted effector protein sopD2 | 142 (51.6) |
| 102 |  | slrP | Type III secretion system effector SlrP, E3 ubiquitin ligase | 119 (43.3) |
| 103 |  | sseK2 | Type III secretion system translocated effectors sseK2 | 119 (43.3) |
| 104 |  | sspH2 | Type III secretion system effector SspH2, E3 ubiquitin ligase | 79 (28.7) |
| 105 |  | sseI/srfH | Secreted effector protein SseI/srfH | 71 (25.8) |
| 106 |  | gogB | Anti-Inflammatory effector gogB | 18 (6.5) |
| 107 | Mg uptake | mgtB | Mg2+ transport protein | 275 (100) |
| 108 |  | mgtC | Mg2+ transport protein | 274 (99.6) |
| 109 | Macrophage inducible genes | mig-14 | Antimicrobial peptide resistance protein, macrophage-inducible gene-14 | 269 (97.8) |
| 110 | Stress protein | sodCI | Gifsy-2 prophage: superoxide dismutase precursor (Cu-Zn) | 68 (24.7) |
| 111 | Gifsy-2 related virulence gene | grvA | Gifsy-2 related virulence gene | 55 (20) |
| 112 | Toxin | cdtB | Cytolethal distending toxin B | 17 (6.2) |
| 113 | Toxin | iucC | Aerobactin synthesis gene | 1 (0.4) |
| 114 | E3 ubiquitin-protein ligase SspH1 | sspH1 | E3 ubiquitin-protein ligase SspH1 | 10 (3.6) |
